# Thermal and Mechanical Properties of Reprocessed Polylactide/Titanium Dioxide Nanocomposites for Material Extrusion Additive Manufacturing

**DOI:** 10.3390/polym15163458

**Published:** 2023-08-18

**Authors:** Saltanat Bergaliyeva, David L. Sales, José María Jiménez Cabello, Pedro Burgos Pintos, Natalia Fernández Delgado, Patricia Marzo Gago, Ann Zammit, Sergio I. Molina

**Affiliations:** 1 Department of Materials Science and Metallurgical Engineering and Inorganic Chemistry, Algeciras School of Engineering and Technology, Universidad de Cádiz, INNANOMAT, IMEYMAT, Ramón Puyol Avenue, 11202 Algeciras, Cádiz, Spain; 2 Physics and Technology Department, Al-Farabi Kazakh National University, 71, Al-Farabi Avenue, Almaty 050040, Kazakhstan; 3 Department of Materials Science and Metallurgical Engineering and Inorganic Chemistry, Universidad de Cádiz, Campus Río S. Pedro, INNANOMAT, IMEYMAT, 11510 Puerto Real, Cádiz, Spain; josemaria.jimenez@gm.uca.es (J.M.J.C.); pedro.burgos@uca.es (P.B.P.); natalia.fernandezdelgado@uca.es (N.F.D.); patricia.marzo@uca.es (P.M.G.); sergio.molina@uca.es (S.I.M.); 4 Department of Metallurgy & Materials Engineering, University of Malta, MSD 2080 Msida, Malta; ann.zammit@um.edu.mt

**Keywords:** material extrusion, additive manufacturing, polylactic acid, recycling, nanocomposite, titanium dioxide

## Abstract

Polylactic acid (PLA) is a biodegradable polymer that can replace petroleum-based polymers and is widely used in material extrusion additive manufacturing (AM). The reprocessing of PLA leads to a downcycling of its properties, so strategies are being sought to counteract this effect, such as blending with virgin material or creating nanocomposites. Thus, two sets of nanocomposites based respectively on virgin PLA and a blend of PLA and reprocessed PLA (rPLA) with the addition of 0, 3, and 7 wt% of titanium dioxide nanoparticles (TiO_2_) were created via a double screw extruder system. All blends were used for material extrusion for 3D printing directly from pellets without difficulty. Scanning electron micrographs of fractured samples’ surfaces indicate that the nanoparticles gathered in agglomerations in some blends, which were well dispersed in the polymer matrix. The thermal stability and degree of crystallinity for every set of nanocomposites have a rising tendency with increasing nanoparticle concentration. The glass transition and melting temperatures of PLA/TiO_2_ and PLA/rPLA/TiO_2_ do not differ much. Tensile testing showed that although reprocessed material implies a detriment to the mechanical properties, in the specimens with 7% nano-TiO_2_, this effect is counteracted, reaching values like those of virgin PLA.

## 1. Introduction

With benefits such as toolless material processing, high geometric freedom, fast prototyping, and cost-efficient small-scale production, additive manufacturing (AM) has the potential to revolutionize the manufacturing industry [1]. Depending on the form of material and the type of extruder, extrusion-based AM can be divided into filament fused fabrication (FFF) and fused granular fabrication (FGF), among others [2]. FFF uses high-quality, not too brittle or too flexible filament with a specific and constant diameter [3]. So, only certain materials with the appropriate mechanical properties can be processed by FFF. In comparison, the FGF method is not so limited by the variety of materials [3], while all industrial polymers can be found as pellets [4]. Using polymeric pellets as a feedstock material can improve production times by up to 200 times [5] and reduce costs by a factor of 10. This is due to the fact that an additional filament-extruding step is not required during the pellet-based AM process [6]. In addition, the one-step preparation of feedstock for FGF excludes a second thermal processing of the polymers, which always reduces their molar mass [3].

Biodegradable polylactic acid (PLA) filaments are one of the most widely used extrusion-based 3D printing feedstocks [3]. PLA is an eco-friendly polymer material [7] based on plant materials [8]. It is a three-carbon-membered thermoplastic with one hydroxyl and one carbonyl at the end. It has a prolonged biodegradation rate and is brittle despite its low degree of crystallinity [9]. However, the role of biodegradable plastics in solid waste management is somewhat controversial because of their slow degradation rate and their possible interference with plastic recycling efforts [10]. Therefore, increasing the production of PLA might cause some problems, mainly related to managing the waste generated after its use [11]. One way to utilize PLA waste is composting [12]. However, this method is used to degrade industrial waste, where a large amount of waste is collected daily [12]. Hence, considering that PLA and the construction of composting facilities are expensive [13,14], reprocessing scrap materials could be an interesting way to save costs [15,16].

Some studies revealed that coupling open-source 3D printers with polymer processing could offer the basis for a new paradigm of the distributed recycling process [1,8,17,18]. The main conclusion from these studies is that 3D printing with recycled PLA is a viable option. A common disadvantage of filament-fed printers reported in these works is nozzle clogging during the reprinting of recycled materials. On the other hand, these studies showed the decreasing tendency of mechanical properties with the addition of recycled content [1,8,17,18]. This limitation can be solved by adding nanofillers, which could also add functionalities to the produced nanocomposites [19]. The physical, chemical, and mechanical improvements are significantly higher than the more traditional polymer composites with micron-sized fillers [20].

Among the many inorganic materials available today, nano-TiO_2_ has received most of the attention [9] because it is nearly non-toxic, inert, optically transparent, biocompatible, environmentally friendly, and inexpensive. Therefore, nano-TiO_2_ has been widely introduced into polymers to improve heat resistance, radiation resistance, mechanical and electrical properties [13], and bacteriostatic and photocatalytic activity [21]. So, the nanoalloy of Ti has a great potential to act as a reinforcing material in PLA composites compared with natural fillers [12]. According to the literature, plentiful dangling bonds exist on the surface of nano-TiO_2_, which could interact with polymer molecules, thus improving the properties of nanocomposites [22]. The literature also revealed that 0.5% to 8% nanofiller reinforcement is sufficient to strengthen the polymer mechanically and thermally. Apart from that, PLA is less susceptible to photodegradation than TiO_2_ nanoparticles; hence, TiO_2_ nanoparticles can improve the photodegradability of PLA [12].

Several investigators have fabricated nanocomposites by reinforcing titanium dioxide in the PLA matrix. The results of Zhuang et al. [10] show that the thermal and mechanical properties are markedly improved when the content of TiO_2_ is 3 wt% in the PLA/TiO_2_ nanocomposites prepared by in situ polymerization. Buzarovska et al. [23] produced nanocomposites with 0.5, 1, 2, 5, and 10 wt% TiO_2_ by solution casting. Zhang et al. [22] employed a vane extruder to compound PLA/TiO_2_ nanocomposites with 0, 0.5, 1.0, 2.0, 5.0, 10.0, and 15.0 wt% TiO_2_ and prepared the samples by injection molding. The prepared nanocomposites showed improved thermal stability for all samples and improved tensile strength in the samples by up to 2%. Nakayama et al. [24] proved that the tensile behavior of PLA films with 10% nano-TiO_2_ was similar to pure PLA. All these studies showed a rising trend in tensile strength when a uniform dispersion of nanoparticles in the matrix of PLA is achieved up to a certain amount of nano-TiO_2_. This upper limit depends on the manufacturing technique, and to the best of our knowledge, nobody has reported the maximum amount of nano-TiO_2_ that produces the best enhancement of the tensile strength on FGF PLA-printed parts. Thus, it can be concluded that the effects of different processing flow fields on the degree of dispersion and the mechanical behavior have not been investigated in detail yet [22]. Also, in 3D printing, mechanical performance depends on the product’s layer adhesion [25], as the bonding strength between two consecutive layers is a weak point of layer-by-layer construction [26].

The aim of this research is to study the possibility of enhancing PLA recyclability by hybridizing reprocessed PLA (rPLA) with virgin PLA and nano-TiO_2_ and using the resulting material as a feedstock for FGF to produce high-quality parts. To achieve this goal, two types of nanocomposite pellets incorporating neat PLA with nano-TiO_2_ and a blend of neat and rPLA with nano-TiO_2_ were prepared. Secondly, samples were FGF-printed from the prepared nanocomposite pellets. This printing technology was preferred because it reduces nozzle clogging during printing and makes filament production unnecessary, saving PLA from additional thermal degradation. Then, the morphology, thermal, and mechanical properties of the produced samples were investigated. Subsequently, the influence of the addition of nano-TiO_2_ and rPLA to neat PLA on the interlayer adhesion of 3D printed samples was analyzed. Finally, the mass fraction of TiO_2_ that improves the mechanical properties of PLA nanocomposites produced by FGF was determined. Although not the focus of this study, the addition of nano-TiO_2_ may also provide further functionalities to the nanocomposites, such as UV resistance and antibacterial activity. Once the feasibility of using the proposed nanocomposites for FGF and their mechanical reinforcement capabilities are proven, this study will serve as a basis for investigating these additional properties of PLA.

The novelty of this work lies in using nanocomposite pellets of PLA and rPLA with the addition of nanoscale titanium dioxide as a feedstock for FGF technology. To the best of our knowledge, there has been no previous report on the preparation and properties studies of 3D printed nanocomposites from PLA with nano-TiO_2_ (PLA/TiO_2_) and from a mixture of PLA and rPLA with nano-TiO_2_ (PLA/rPLA/TiO_2_).

## 2. Materials and Methods

Six mixtures from neat PLA pellets with or without adding rPLA and/or titanium dioxide nanoparticles were produced for investigation and comparison. Proportions of PLA and rPLA pellets and nano-TiO_2_ are presented in Table 1. The designations of the different composites are as follows: letter V stands for virgin PLA, R for one-time reprocessed PLA, and A for additives, i.e., titanium dioxide nanoparticles. The number to the right of the letter indicates the percentage of each material in the mixture.

### 2.1. Materials

PLA granules named NatureWorks 3D850, purchased from NatureWorks LLC (Plymouth, MI, USA), were used as virgin PLA, with a specific gravity of 1240 kg/m^3^, a relative viscosity of 4.0, peak melt temperature of 165–180 °C, and 55–60 °C glass transition temperature, as reported in the manufacturer’s technical data and security sheet [27]. An additive nanopowder of titanium (IV) oxide with a particle size of approximately 10–20 nm was purchased from ALDRICH Chemistry (Taufkirchen, Germany) and used as received.

### 2.2. Production of Reprocessed PLA

Reprocessed PLA was obtained under simulated recycling conditions by melt-processing virgin PLA in an extruder system to emulate recycled PLA [8,17]. About 700 g of raw PLA granules were dried overnight at 50 °C to remove residual moisture in a Piovan DPA 200 (Group Piovan, Maria di Sala VE, Italy) dehumidifying system. Then, this material was processed in a twin-screw modular extruder system by Scamex (Isques, France). It has five heater zones with controlled temperatures, screw speed, and work pressure, and two electronic dosimeters with different blades: the first for pellets and the second for powders. A continuous filament with a diameter of 1.75 mm was produced. The diameter of the filament was automatically adjusted by using the optical reader of the winder. This filament was automatically cut into small pieces in the extruder, which were then used as the reprocessed part in the composites. The technical data used for processing the samples is shown in Table 2. The mixture of virgin and rPLA V25R75 was produced by extruding the proportion of 25% virgin PLA and 75% rPLA.

### 2.3. Manufacturing of Nanocomposites

**Figure 1:**
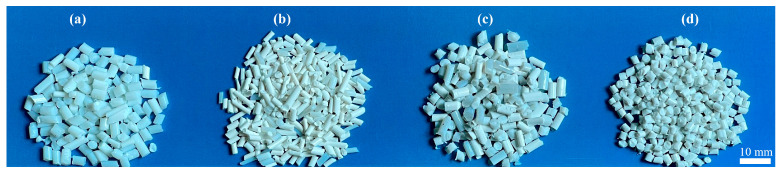
Pellets from different nanocomposites: (**a**) V97A3; (**b**) V93A7; (**c**) V22R75A3; (**d**) V18R75A7.

To produce each composite material, V97A3, V93A7, V22R75A3, and V18R75A7, PLA was introduced in the extruder by the first dosimeter and the TiO_2_ nanoparticles by the second dosimeter. Table 2 presents the processing conditions. Figure 1 shows pictures of the pellets produced.

### 2.4. Printing

**Figure 2:**
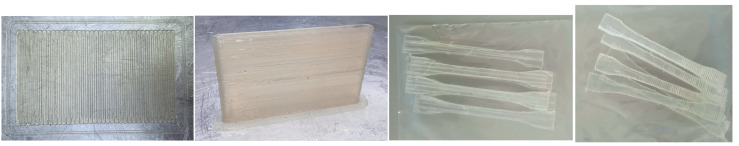
Horizontal and vertical plates of size 150 × 90 × 2 mm (first two on the right, respectively) and examples of specimens for tensile tests in the XY and XZ orientations (last two on the left, respectively).

Specimens were printed using direct pellet extrusion technology with the Discovery 3D Granza printer from Bárcenas CNC (Valdepeñas, Ciudad Real, Spain); its printing volume is 1100 × 800 × 500 mm. The slicer software Simplify3D (Simplify3D, Cincinnati, OH, USA) was used to prepare the files in a G-code format for printing out the specimens. Table 3 shows the printing parameters used. For horizontal specimens, a 100% linear infill at 0° (XY orientation) was used, and for vertical specimens (XZ orientation), a unique contour (nomenclature according to AM standard [28]) was used. All compositions processed with FGF were dried for 4 h at 60 °C in a Piovan DPA (Group Piovan, Maria di Sala VE, Italy) dehumidifying dryer to avoid possible defects due to humidity. Pictures of the resultant horizontal and vertical FGF-printed samples are shown in Figure 2.

The temperature of the extruder (namely the three heating zones of the extruder, the last one of which is the closest to the nozzle), the temperature of the bed, and the printing speed for horizontal and vertical plates were constant. Table 4 lists the parameters used to manufacture the plates. The temperatures have been selected according to the PLA manufacturer’s recommendations for printing (around 200–220 °C) and to previous prints carried out by the authors on the used printer. The temperature gradient of the extruder (with increases of 5 °C per part) was set according to the recommendation of the printer manufacturer and its technical characteristics. The multiplier is a parameter that controls the rate of extruded material and is experimentally set. The variations in the used multiplier value are due to the differences in the rheological behavior of the blends, which depend on the additive content. This correction aims to maintain a constant flow throughout the printing process and to be able to manufacture plates with perfectly joined beads. The multiplier of each composition is varied for the vertical sheet with respect to the horizontal one to preserve the extrusion width of 2 mm. Finally, the printing speed for horizontal plates is the one recommended by the printer manufacturer for printing standard parts, and it was reduced for the vertical specimens, so the filament has enough time to adhere to the previous layer, hence avoiding sobbing.

Considering the multiplier for a PLA material of 0.2, the flow of V18R75A7 is moderately controllable, somewhat better than for the V100, V97A3, and V22R75A3. Therefore, the plate comes out quite full. In the case of horizontal and vertical plates from V22R75A3, it was only necessary to increase the multiplier to 0.22 to cover the plate well. The V93R7 is a compound that needed the least multiplier of 0.18 due to its high fluidity at the temperature range used. However, there is a problem with some flow control, which was also experienced in other compounds. In contrast, the V25R75 compound requires much more multiplier extrusion than the rest because of the 75% of rPLA, considering the parameters used in the rest of the materials, raising the multiplier to 0.26. Finally, the V100 compound performs very well in printing, as it is an untreated and pure base, avoiding excess material and flow variations during printing. On the vertical plate, excellent wall stability is observed, as expected. It must be mentioned that warping, cracking, delamination problems, or nozzle clogging during the 3D printing process were not detected in any sample.

### 2.5. Cutting of Samples

The specimens were cut from the FGF-printed sheets to the required dimensional accuracy. At least five tensile specimens of type 1BA (i.e., a reduced-size version of probes extracted from machining) according to ISO 527-2 [29] were milled with a LEKN(C1) 3020 CNC Router Machine Kit (Lekn, Nanjing, China), using a 2 mm diameter flat milling cutter with two cutting edges. A milling speed of 5000 rpm and a cutting speed of 350 mm/min were used for both horizontal and vertical printed plates. Before milling, the surfaces of the plates were covered with an adhesive film to prevent the plate from being overcoated and the chips resulting from the milling process from sticking to it.

### 2.6. Characterization and Testing

Following tensile testing, the fractured surface was examined via scanning electron microscopy (SEM). This test was conducted to show the distribution of nanoparticles in polymer matrix. SEM measurements were carried out using an FEI Nova NanoSEM 450 (Fei, Waltham, MA, USA) microscope with a field-emission gun for high-resolution analyses controlled by xT Microscope Server software (Fei, Waltham, MA, USA). Secondary electron detectors with 5 kV, a probe size of 2.0 nm, and magnifications of 40X and 20 kX were used. To carry out the EDX analysis, an EDAX detector and the AZtec software from Oxford Instruments (Abingdon, UK) were used. To protect the samples during the analysis, they were covered by a 10 nm layer of gold using a Balzers SCD 004 Sputter Coater (Balzers, Liechtenstein). SEM images were analyzed and processed using ImageJ software (National Institute of Health, Bethesda, MA, USA) [30].

Differential scanning calorimetry (DSC) experiments were performed in a Q20 (T&A Instruments, Austin, TX, USA) according to ISO 11357-1 [31]. Temperature sweeps were performed from room temperature to 200 °C at 10 °C/min under nitrogen flow. The glass transition, melting and crystallization temperatures, and degree of crystallization (T_g_, T_m_, T_c_, and X_c_, respectively) were determined by the heating process.

Thermogravimetric analysis (TGA) was carried out in a Q50 (T&A Instruments, Austin, TX, USA) in accordance with ISO [32]. Samples of approximately 10 mg of each polymer/blend were tested. A temperature sweep was performed from room temperature to 600 °C at 10 °C/min under nitrogen flow.

Tensile testing of the printed specimens was performed on a universal testing machine (Shimadzu, Kyoto, Japan) at a constant speed of 1 mm/min, according to ISO 527-1 [28]. At least five specimens were tested for each material. The Young’s modulus, tensile strength, and elongation at break values were determined for each specimen. Results were averaged, and standard deviations were presented as error bars.

## 3. Results

### 3.1. Scanning Electron Microscopy

**Figure 3:**
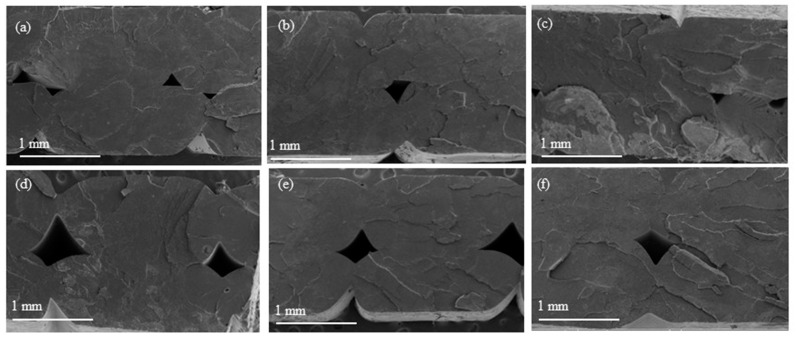
SEM images showing the topography of fracture surfaces of the FGF printed samples: (**a**) V100; (**b**) V97A3; (**c**) V93A7; (**d**) V25R75; (**e**) V22R75A3; (**f**) V18R75A7. Flat surfaces with small steps, typical of brittle fracture, are shown in all samples. The deposited beads can also be distinguished.

The dispersion of nanoparticles in the polymer matrix is a crucial factor influencing the physical properties of the nanocomposites. Therefore, the SEM analysis of the FGF-printed PLA, PLA/TiO_2_, and PLA/rPLA/TiO_2_ nanocomposites was performed on fracture surfaces of post-tested tensile test samples (Figure 3), to investigate the dispersion and distribution of TiO_2_ nanoparticles within the biodegradable matrix. The SEM images do not show any significant differences at a magnification of 40 k. The surfaces are flat and smooth, indicating a brittle nature, consistent with the break without necking observed in the tensile tests (see Section 3.4) and a similar study by Thumsorn et al. [25].

**Figure 4:**
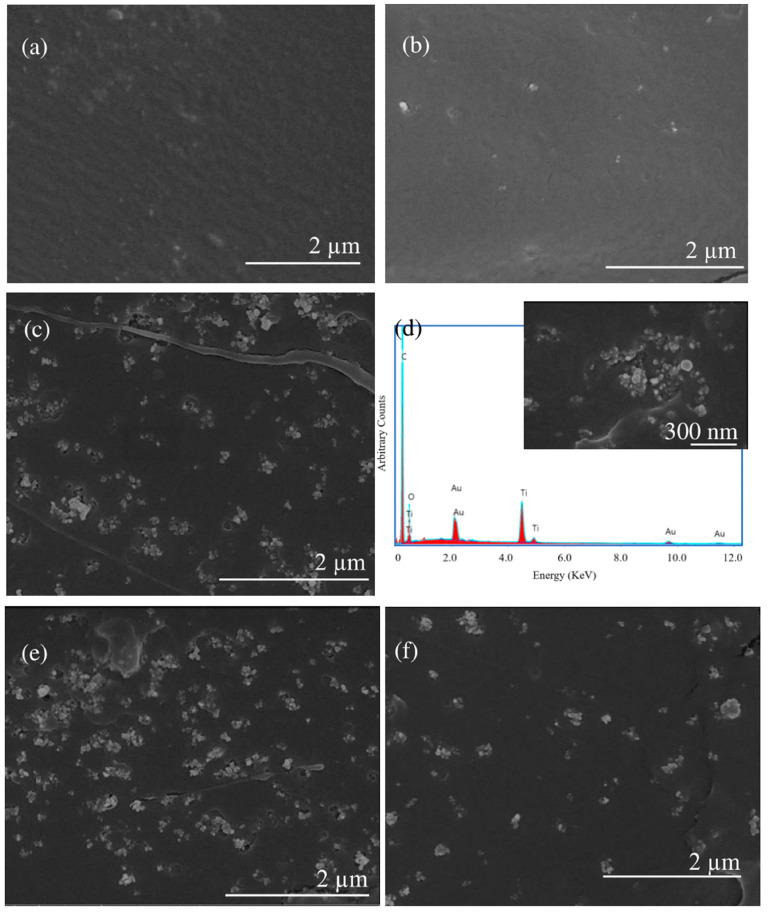
TiO_2_ nanoparticles and their aggregates are shown with a brighter contrast in the secondary electron SEM images. (**a**) SEM image of V100 sample; (**b**) SEM images of V97A3 sample; (**c**) SEM image of V93A7 sample; (**d**) EDX spectra of one of the V93A7 zones with TiO_2_ particles (the inset is an image of the region of the sample where EDX was taken); (**e**) SEM image of V22R75A3 sample; (**f**) SEM image of V18R75A7 sample.

The SEM analysis was also carried out at higher magnifications to study the integration of the nanoparticles in the polymer. As shown in Figure 4, the TiO_2_ nanoparticles are revealed with a higher intensity in the secondary electron micrographs and are clearly differentiated from the PLA matrix. EDX analysis was carried out to corroborate the composition of the TiO_2_ nanoparticles. Figure 4d shows the Ti peak, corroborating the presence of TiO_2_. The Au peak is due to the gold coating necessary for SEM analysis of organic samples (see Section 2.6).

**Figure 5:**
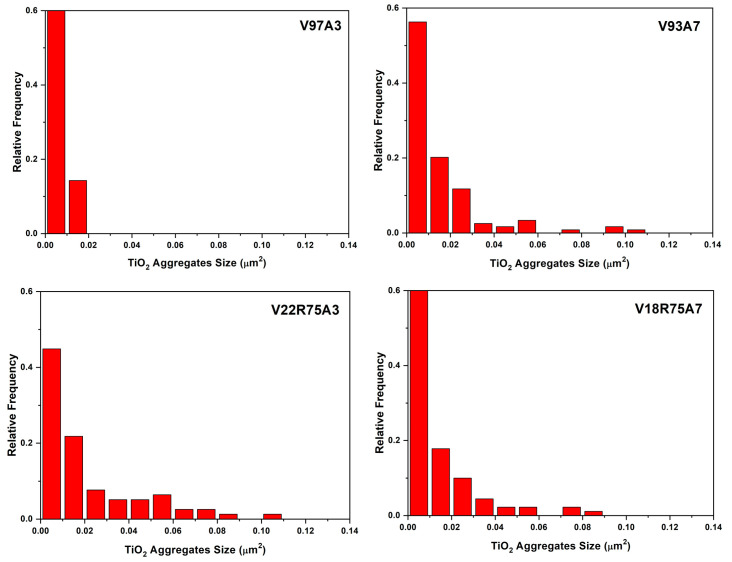
Normalized frequencies of TiO_2_ aggregates.

Images in Figure 4 show a homogeneous distribution and adequate integration of the TiO_2_ in all samples. Nevertheless, Figure 4c,e,f depict that the nanoparticles are gathered into agglomerations in V93A7, V22R75A3, and V18R75A7. The areas corresponding to TiO_2_ were measured on the high-magnification images to know the equivalent diameters of nanoparticle agglomerations. The histogram of aggregates’ equivalent diameter is presented in Figure 5. The areas of agglomerations range from 0.0003 µm^2^ to 0.2180 µm^2^. According to the manufacturer, the average size of TiO_2_ nanoparticles is between 10 and 20 nm. Considering this information, the equivalent diameter of the smallest agglomeration with an area of 0.0003 µm^2^ is around 20 nm. Then, the smallest bright areas in the received micrographs consist of one TiO_2_ nanoparticle. Isolated nanoparticles can be seen in all samples, but their density is low for all samples (<7 × 10^6^ cm^−2^). Otherwise, the percentage of 2-nanoparticle aggregates is higher than the one for separate particles and is 14, 14, 13, and 10% in V97A3, V93A7, V22R75A3, and V18R75A7, respectively. The biggest agglomeration was revealed in V93A7, with an area of 0.2180 µm^2^ corresponding to the union of 26 nanoparticles of 20 nm diameter. The results of measured equivalent diameters were statistically normalized in OriginPro software (OriginLab, MA, USA) and presented in Figure 5. The agglomeration sizes that presented more frequency are 0.003 µm^2^ and 0.005 µm^2^ (about 3–4 nanoparticles of 20 nm diameter) for V97A3, 0.01 µm^2^ (about 5–6 nanoparticles with size 20 nm) for V93A7 and V22R75A3, and 0.005 µm^2^ (4 nanoparticles of 20 nm diameter) for V18R75A7. From this data, it can be estimated that the highest content of agglomerations consists of 3–6 nanoparticles in all samples. Figure 5 illustrates that V18R75A7 has smaller agglomeration sizes than V93A7 and V22R75A3.

The tendency to aggregate can be explained by the fact that no surface treatment was performed on the oxide particles, as in the study in reference [23]. Severe aggregation of TiO_2_ nanoparticles could be reduced by surface modification using carboxylic acid and long-chain alkyl amine, as Nakayama et al. did [24]. The gathering of nanoparticles in agglomerations may be due to the hydrogen bonds on the surface of the TiO_2_ particles. Dubois et al. [33] and Zhuang et al. [10] stated that, because of the unique surface properties of the nanoparticles, they easily formed both soft and hard agglomeration. Electrostatic forces and Van der Waals forces mainly cause soft agglomeration. These forces are weak, and this agglomeration can be eliminated through chemical or mechanical processes. By contrast, hard agglomeration is caused by many kinds of forces, including Van der Waals forces, Coulomb forces, and chemical bonding. As a result, the particles are closely combined, and it is not easy to eliminate this kind of agglomeration.

Therefore, SEM micrographs revealed good dispersion of nano-TiO_2_ aggregates in the matrix at low nano-TiO_2_ content. In contrast, higher content contributed to aggregation within the matrix, which was consistent with the results of mechanical and thermal properties. The same results were reported by Zhang et al. [13].

### 3.2. Thermogravimetric Analysis

**Figure 6:**
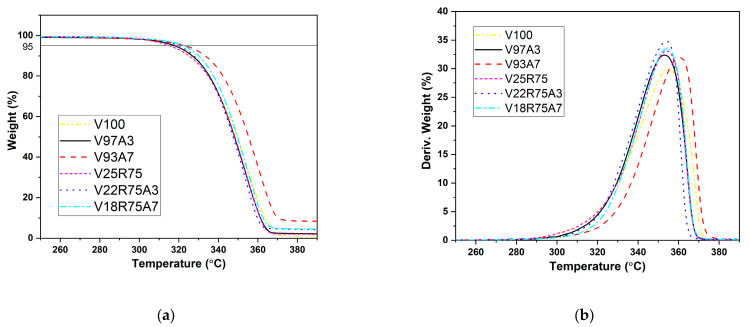
(**a**) Thermogravimetric curve of PLA and its nanocomposites; (**b**) Respective derivative thermogram curves.

The effect of nano-TiO_2_ addition on the thermal stability of PLA nanocomposites was evaluated by thermogravimetry (TG). Figure 6 illustrates the TG curves and their respective derivative thermograms (DTG) of pure PLA, the mixture of pure and rPLA, and their nanocomposites. TGA curves of PLA nanocomposites have a single-stage sample weight reduction with a maximum decomposition temperature (T_max_) of around 350 °C. T_max_ is listed in the last row of Table 5 (extracted from DTG in Figure 6b), evidencing that the composites degrade similarly to PLA.

V100 has the lowest decomposition temperature at 5% weight loss (T_5%loss_) among all samples. This temperature rises with the addition of rPLA, V25R75, experiencing an increase of almost 3 °C. This effect is also observed after adding nano-TiO_2_, so the T_5%loss_ of PLA/TiO_2_ and PLA/rPLA/TiO_2_ nanocomposites shifted to a higher value than the reference samples without TiO_2_. This fact indicated that the addition of nano-TiO_2_ improved the thermal stability of nanocomposites. Generally, the particles can enhance the thermal stability of a polymer because the presence of nano-TiO_2_ particles constrains the mobility of PLA molecular chains [10,13].

The temperature T_max_ shows the maximum degradation temperature. According to the results presented in Figure 6 and Table 5, most of the samples, except V93A7, reached maximum degradation conditions at the same temperature range of about 353–355 °C. For polymers without nanoparticles, complete degradation occurs at about 400 °C. With nanoparticles, zero residue weight was not reached when the samples were heated up to 600 °C. Hence, the particles are stable in the considered temperature range. TGA results show that introducing TiO_2_ has a rising tendency to increase thermal stability for both PLA/TiO_2_ and PLA/rPLA/TiO_2_ nanocomposites with increasing nanoparticle concentration. This agrees with the results of Zhang et al. [22], who showed that introducing TiO_2_ significantly improved thermal stability.

### 3.3. Differential Scanning Calorimetry

**Figure 7:**
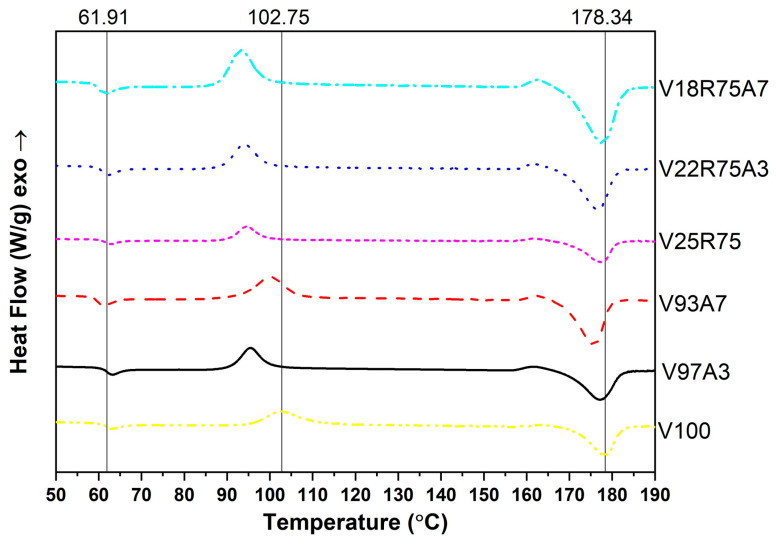
Differential scanning calorimetry curves.

Table 6 and Figure 7 illustrate the results of the DSC test. They show that T_g_ appears to have a value between 60 and 62 °C and does not change much from sample to sample. This conclusion also applies to T_m_. Otherwise, T_c_ is higher for virgin PLA samples than rPLA samples. The reduction of T_c_ can be attributed to the higher mobility of the polymer chain due to the reduced molecular weight in V25R75 and their nanocomposites [33].

The degree of crystallinity X_c_ was quantified according to [34,35,36,37] as:

X_c_ = (∆H_m_ − ∆H_c_)/∆H∗ × 100 (1)

where ΔH∗ = 93 J/g and denotes the enthalpy of fusion for an infinitely large crystal [8].

Comparing the crystallinity degree X_c_ of V100 and V25R75, the higher crystallinity of the PLA mixture can be noticed. This could be attributed to the fact that the recycled part of V25R75 has molecules with shortened molecular chains, as was mentioned earlier, that can organize crystals easier. For both groups of nanocomposites, PLA and rPLA, there is a clear trend: the reduction in crystallinity when increasing the nanoparticle concentration. As mentioned in Section 3.1, nanoparticles form agglomerations, which can restrict the mobility of PLA macromolecules and the formation of crystals. In this study, the TiO_2_ nanofiller has no significant influence on the T_g_ and T_m_ temperatures but affects the mobility of macromolecular chains in all investigated samples. Similar results were observed by [22,23,38].

### 3.4. Tensile Testing

In this study, all samples were printed in both horizontal and vertical orientations. The tensile strength of V100 is 55.100 ± 2.243 MPa, which is higher than that of V25R75, which is 48.535 ± 2.590 MPa in horizontal printed samples. The reduction in tensile strength after 3D printing reprocessing was also observed by Anderson [8] and Cruz Sanchez et al. [17]. This can be explained by the tendency of PLA to undergo degradation during thermal processing from the molten state, giving a rapid reduction of molecular weight [39], which was seen in Section 3.3.

Nanocomposites from 100% rPLA were not produced because previous experiments conducted with 100% rPLA samples (not considered in this study) resulted in a drastic drop in the vertical tensile strength of almost half of that for V100, from 36.242 ± 2.512 MPa to 18.675 ± 0.711 MPa. In contrast, the tensile strength in the vertical direction of V25R75 stayed almost the same as V100. This was the reason for considering TiO_2_ nanocomposites with a maximum content of 75% rPLA in this work. This difference in the tensile strength of rPLA and V25R75 can be explained by the fact that the strength acquired by the vertical specimen can be considered to be due to the adhesion strength between layers [40]. The strength of printed parts depends on the strength of the used thermoplastic filament and the bond strength between layers [41]. Thus, it can be concluded that short molecular chains in rPLA cannot form strong interlayer adhesion during printing compared with V25R75 with long and short flexible molecular chains, which have more robust molecular entanglement.

**Figure 8:**
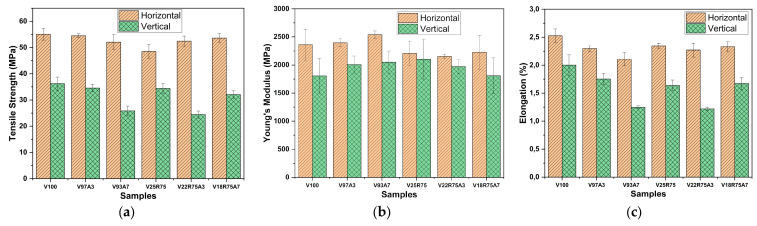

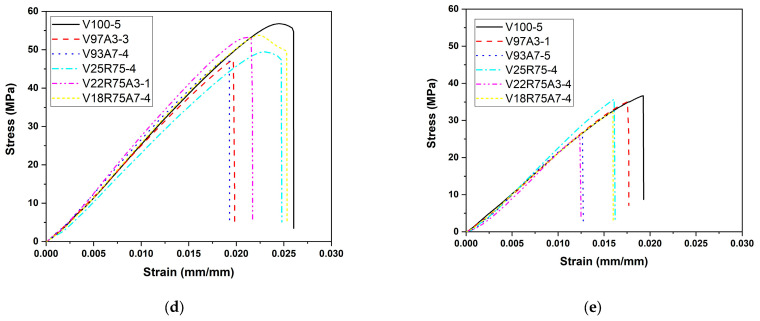
Results of the tensile tests. Average values with error bars of the main mechanical properties: (**a**) tensile strength, (**b**) Young’s modulus, and (**c**) elongation. Strain-stress curves of one of the specimens per batch of (**d**) the horizontal samples and (**e**) the vertical samples.

The results presented in Figure 8 show that the addition of nanoparticles to pure PLA reduces the tensile strength in both printing directions. Zhang et al. [22] revealed that adding nano-TiO_2_ to injection-molded samples up to 2 wt% slightly shifts tensile strength to a higher value than pure PLA. However, when the TiO_2_ content is greater than or equal to 2 wt%, the nanocomposites show a lower tensile strength than neat PLA. At high loading, the lack of strong interaction between polymer and particles due to filler aggregation resulted in debonding of the particles at lower tensile stress and a subsequent premature yield [14]. So, it can be said that adding 3–7% of nanoparticles to pure 3D-printed PLA is too much to enhance the mechanical properties of nanocomposites. This is due to the nano-TiO_2_ agglomerations, seen in Section 3.2, which restrict the movement of the polymer chains in the composites.

On the other hand, positive dynamics can be observed due to the addition of nanoparticles to a mixture of pure and reprocessed polymers. The tensile strengths of V22R75A3 and V18R75A7 increase from 48.535 ± 2.590 MPa to 52.470 ± 1.916 and 53.622 ± 1.651 MPa in the horizontal printing direction, respectively. These results show that the tensile strength in XY orientation of nanocomposites from the mixture of virgin and rPLA reaches the same value as V100. In the vertical direction, the tensile strength of V18R75A7 is almost the same as V25R75, considering the standard deviation. Hence, nanocomposite from PLA and rPLA with 7% TiO_2_ has almost the same tensile strength as specimens manufactured through FGF from 100% PLA. The formation of two new bonds can explain it. First, according to the published research, there are many hydroxyl groups (Ti–OH) covering the surface region of TiO_2_ nanoparticles, which could form a strong interfacial bond (Ti–O–C) with the carbonyl groups of PLA [22]. In this sense, compared with V100, V25R75 would have increased the concentration of carboxylic acid end groups in the degradation medium because of chain scission in recycled PLA [39]. Secondly, Zhang [13] reported the increasing number of hydrogen bonds being formed between the titanium hydroxyl and hydroxyl groups of the PLA matrix. That is why it can be concluded that the molecular chains of the mixture with rPLA have more hydroxyl and carbonyl end groups that can form strong internal friction (interaction) between nanoparticles in the matrix of V18R75A7, hence enhancing tensile strength. Also, it was mentioned in Section 3.1 that V18R75A7 has a lower agglomeration size than V93A7 and V22R75A3, which may be the reason for its slightly higher strength.

According to Figure 8b, the Young’s Modulus of V97A3 and V93A7 rises when increasing the TiO_2_ content in both printing directions, while this effect is not observed in nanocomposites with rPLA. Considering the standard deviation, the Young’s Modulus of nanocomposites from neat and rPLA is approximately the same as the reference sample in both XY and XZ orientations. It must be mentioned that V93A7 has the highest value of Young’s Modulus among all the studied samples.

The values of elongation presented in Figure 8c follow a similar tendency as the tensile strength for all the samples, with the only exception of the horizontal rPLA sample series, in which ductility does not vary with the addition of TiO_2_. Therefore, ductility is generally reduced when adding nanoparticles, making them more brittle.

## 4. Conclusions

This study investigates the preparation and the thermal, mechanical, and structural characterization of six polymer blends for FGF additive manufacturing: pure PLA, a mixture of 25% pure and 75% reprocessed PLA, and their nanocomposites with 3 and 7% TiO_2_. From the derived results, the following can be concluded:

- Regarding the manufacturing of the granules, the extruder’s technical setup varies for every granule type and is experimentally established. The screw speed varies from 130 rpm for a mixture of 25% PLA and 75% rPLA to 100 rpm for nanocomposites based on rPLA.
- The printing parameters were established experimentally too. The multiplier values for horizontal and vertical printed samples change from 0.16 for PLA to 0.27 for the mixture of PLA and rPLA. Other printing parameters were constant.
- All granules showed good flowability and printable quality.
- Even though all blends showed nanoparticle agglomerations, they were uniformly distributed.
- The thermal stability has a rising tendency when increasing the additive’s content. The T_5%loss_ rises by 10 °C for nanocomposites with 7% TiO_2_ compared with PLA.
- The crystallization temperature and degree of crystallinity of nanocomposites decreased with the addition of TiO_2_. For example, PLA has a degree of crystallinity of about 15%, the nanocomposite with 3% TiO_2_ is 11%, and the one with 7% TiO_2_ is 9%. The same tendency can be seen for samples with reprocessed PLA, so the mixture of PLA and rPLA has a degree of crystallinity of about 20%, the nanocomposite with 3% TiO_2_ is 15%, and the one with 7% TiO_2_ is 10%.
- Tensile testing showed that adding nanoparticles to pure PLA reduces the tensile strength and increases the Young’s Modulus in both printing directions. However, this effect is not observed in nanocomposites with rPLA. Nanocomposite from primary and secondary PLA with 7% nano-TiO_2_ has almost the same mechanical characteristics as PLA.

In summary, FGF nanocomposites based on a blend of virgin and recycled PLA with titanium dioxide nanoparticles are excellent options for improving recycled PLA’s tensile strength and thermal stability and adding functionalities to the material. Future research will be aimed at checking the resistance of the produced nanocomposites to UV degradation and their antibacterial activity.

## Figures and Tables

**Table 1:** Sample name and composition. Letter V stands for virgin polylactide, R for one-time reprocessed polylactide, and A for additives, i.e., titanium dioxide nanoparticles.

Sample Designation	Weight Ratio (%)		
	Virgin Polylactide	Reprocessed Polylactide	Nanoparticles of Titanium Dioxide
V100	100	0	0
V97A3	97	0	3
V93A7	93	0	7
V25R75	25	75	0
V22R75A3	22	75	3
V18R75A7	18	75	7

**Table 2:** Technical data to manufacture reprocessed polylactide, the mixture of neat and reprocessed polylactide, and nanocomposites.

Sample Code	Central Unit						Dehumidifier	Dosimeter		Winder	Pelletizing Machine		Work Pressure (Bar)
	Screw Speed (rpm)	T1 (°C)	T2 (°C)	T3 (°C)	T4 (°C)	T5 (°C)	Initial Material Temperature (°C)	D1 (rpm)	D2 (rpm)	Winder Speed (m/min)	Cutting (rpm)	Pull (rpm)	
rPLA	126	180	180	190	200	200	60	2.5	-	2.3	44	32	3
V97A3	130	185	190	190	180	175	60	1.5	3	1.75	27	20	7
V93A7	130	185	190	190	180	175	60	0.5	6	1.75	27	20	7
V25R75	130	185	190	190	180	175	60	1.5	3	1.75	27	20	8
V22R75A3	100	185	190	190	180	185	60	1.5	5	1.20	27	20	7
V18R75A7	100	185	190	190	180	185	60	1.5	5	1.20	48	18	8

**Table 3:** Three-dimensional printing parameters.

Nozzle Diameter (mm)	Bead Width (mm)	Layer Height (mm)
2	2	1

**Table 4:** Fused granular fabrication printing parameters.

Composite	Multiplier Horizontal Specimens	Multiplier Vertical Specimens	Temperature of Extruder (°C)	Temperature of the Bed (°C)	Print Speed Horizontal Specimen (mm/s)	Print Speed Vertical Specimen (mm/s)
V100	0.16	0.16	205/210/215	50	50	23
V97A3	0.22	0.22				
V93A7	0.18	0.20				
V25R75	0.26	0.27				
V22R75A3	0.22	0.22				
V18R75A7	0.23	0.24				

**Table 5:** The results of the thermogravimetric analysis: decomposition temperature at 5% weight sample loss and maximum decomposition temperature (T_5%loss_ and T_max_, respectively). All values are given in °C.

Sample Code	V100	V97A3	V93A7	V25R75	V22R75A3	V18R75A7
T_5%loss_	312.000	317.803	322.191	315.117	319.000	322.000
T_max_	355.114	352.859	361.042	354.037	353.930	354.227

**Table 6:** Results of DSC tests: glass transition, crystallization, and melting temperatures (T_g_, T_c_, and T_m_, respectively) are shown, as are the enthalpies of crystallization and fusion (∆H_c_ and ∆H_m_) and the calculated degree of crystallinity (X_c_).

Sample Code	T_g_ (°C)	T_c_ (°C)	∆Hc (J/g)	T_m_ (°C)	∆H_m_ (J/g)	X_c_ (%)
V100	61.91	102.75	31.83	178.34	46.08	15
V97A3	62.16	95.57	31.25	177.18	41.62	11
V93A7	60.19	99.97	37.34	175.66	46.03	9
V25R75	62.57	94.69	26.61	177.29	45.02	20
V22R75A3	60.86	93.96	29.15	176.54	43.22	15
V18R75A7	60.25	93.09	36.43	177.67	44.66	10

## Data Availability

Data are available upon reasonable request by contacting the corresponding author.
